# Realistic boundary conditions in SimVascular through inlet catheter modeling

**DOI:** 10.1186/s13104-021-05631-7

**Published:** 2021-05-31

**Authors:** Amirtahà Taebi, Selin Berk, Emilie Roncali

**Affiliations:** 1 Department of Biomedical Engineering, University of California, Davis, One Shields Ave, Davis, CA 95616-5270 USA; 2 Department of Mechanical and Aerospace Engineering, University of California, Los Angeles, 420 Westwood Plaza, Los Angeles, CA 90095-1597 USA; 3 Department of Radiology, University of California, Davis, 4860 Y Street, Suite 3100, Sacramento, CA 95817 USA

**Keywords:** SimVascular, Inlet boundary condition, Catheter presence, Computational fluid dynamics

## Abstract

**Objective:**

This study aims at developing a pipeline that provides the capability to include the catheter effect in the computational fluid dynamics (CFD) simulations of the cardiovascular system and other human vascular flows carried out with the open-source software SimVascular. This tool is particularly useful for CFD simulation of interventional radiology procedures such as tumor embolization where estimation of a therapeutic agent distribution is of interest.

**Results:**

A pipeline is developed that generates boundary condition files which can be used in SimVascular CFD simulations. The boundary condition files are modified such that they simulate the effect of catheter presence on the flow field downstream of the inlet. Using this pipeline, the catheter flow, velocity profile, radius, wall thickness, and deviation from the vessel center can be defined. Since our method relies on the manipulation of the boundary condition that is imposed on the inlet, it is sensitive to the mesh density. The finer the mesh is (especially around the catheter wall), the more accurate the velocity estimations are. In this study, we also utilized this pipeline to qualitatively investigate the effect of catheter presence on the flow field in a truncated right hepatic arterial tree of a liver cancer patient.

**Supplementary Information:**

The online version contains supplementary material available at 10.1186/s13104-021-05631-7.

## Introduction

Finite element analysis is a powerful method to investigate blood flow behavior inside the circulatory system toward early diagnosis and treatment efficiency evaluation. To accurately capture the hemodynamics details using finite element simulations, it is necessary to impose realistic patient-specific conditions on the boundaries of the computational domain. For example, previous studies showed that inlet velocity conditions significantly affect the flow field downstream of the inlet [1].

**Fig. 1:**
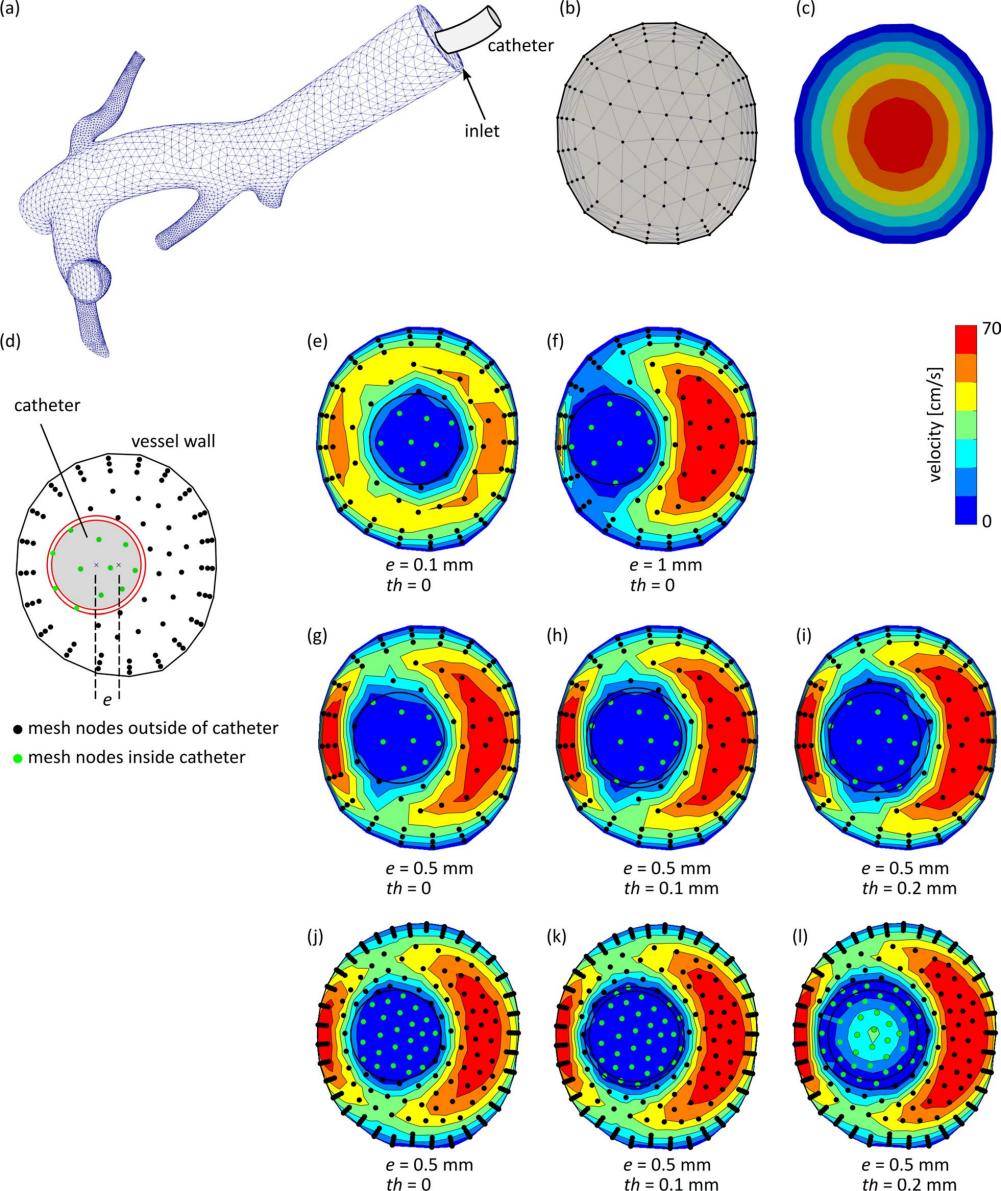
**a** Computational domain, **b** mesh nodes in the inlet plane, **c** parabolic velocity profile available in SimVascular, **d** catheter design, **e**–**i** resulting velocity distribution in the presence of a catheter with different catheter eccentricity (*e*), wall thickness (*th*), flow profile, and mesh density. In all cases, the vessel and catheter flows are forward flow, i.e. going into the computational domain

The open-source software SimVascular [2] has been utilized for image-based flow modeling inside the body for various applications [3–6]. The inlet boundary conditions currently available in the software package include “prescribed velocities” where users can choose between parabolic, plug, or Womersley velocity profiles. Although these analytical shapes can properly model the inlet conditions in many hemodynamics studies, they cannot appropriately estimate the inlet velocity profile when a catheter is inserted into the vessel (Fig. 1a). Catheters are commonly used in medical procedures in a wide range of applications from drug delivery such as in tumor embolization to dialysis, injection of intravenous fluids, and invasive methods of measuring cardiovascular parameters. Previous studies suggest that the presence of a catheter affects the local flow field and flow distribution in downstream vessel trees [7–10] and therefore it might alter the hemodynamics quantities of interest [11, 12]. For example, when a catheter is used to administer antibiotics, bland particles, chemo beads, glue (as an embolism material), parental nutrition, or other drugs in liquid form, any alterations in the flow field, even in the short period of time that the catheter is inserted into the vessels, can change the downstream blood flow and therapeutic agent distribution, and consequently affect the procedure efficiency. Thus, it is essential to account for the catheter presence in the inflow boundaries to achieve physiologically realistic cardiovascular simulations. In this study, we develop and present a pipeline that maps a volumetric flow rate of interest to the inlet plane, which contains a catheter with an adjustable radius, wall thickness, and eccentricity. The pipeline output is a boundary condition file compatible with the software SimVascular. This can help improve the accuracy of the simulation results in the applications that there is a catheter in the vessel such as interventional radiology procedures (See the “Sample application” section for an example in liver cancer radioembolization).

## Main text

### Methods

The objective of this study was to develop an open-source code to help simulate the presence of the catheter in computational fluid dynamics (CFD) simulations performed by SimVascular considering the options and physics included in its current version (September 2020). Under these circumstances, the effect of the catheter was approximated by manipulating and modifying the boundary condition file generated by SimVascular for the simulation rather than modeling the catheter as a part of the computational domain.

**Fig. 2:**
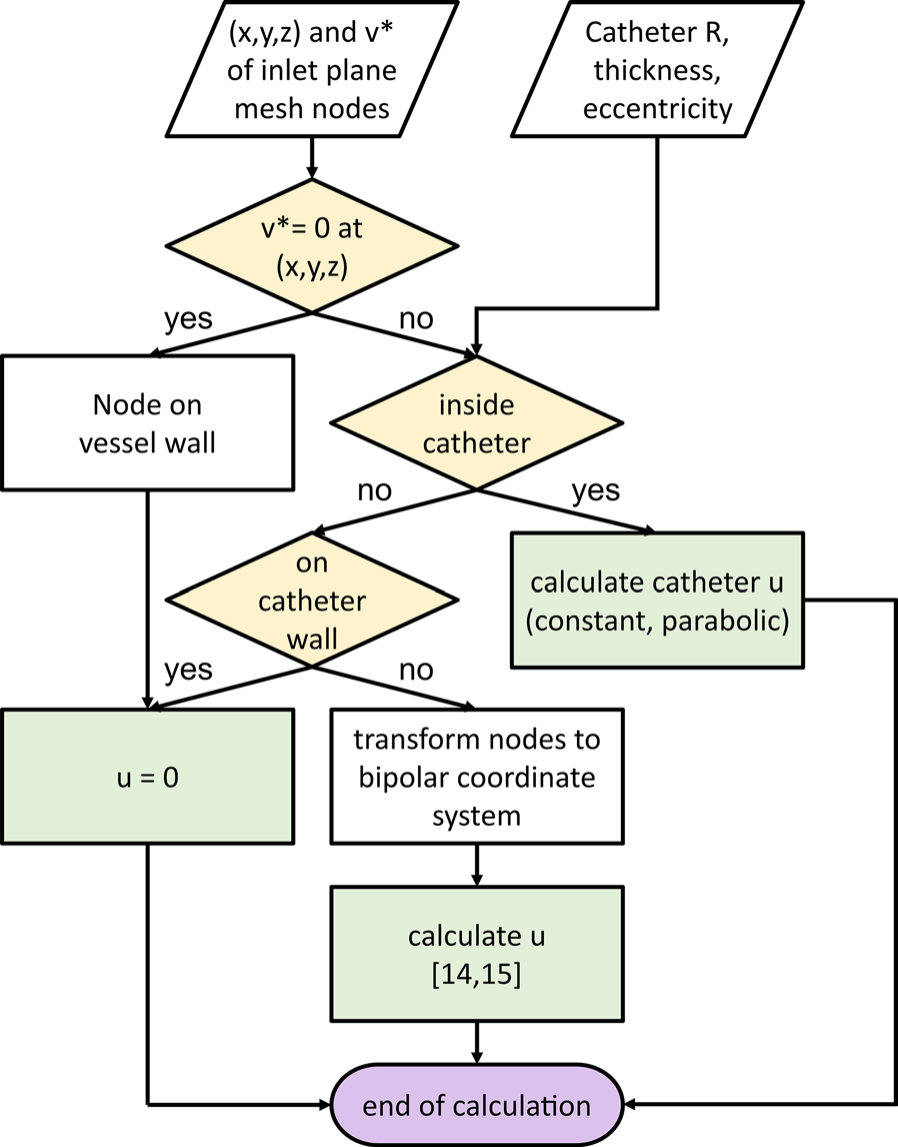
Flowchart for calculating velocity distribution *u* in the inlet cross-section in the presence of a catheter, modeled through its thickness, eccentricity, and radius (R)

MATLAB (R2018b, The MathWorks, Inc., Natick, MA, USA) was used to calculate the velocity profiles and generate the boundary condition files (bct.dat) for the SimVascular simulations (Codes are available in[13]). Figure 2 shows a flowchart of the sequence of the steps to calculate the inlet velocity profile. In this figure, (x,y,z) are the Cartesian coordinates of the mesh nodes in the inlet plane (Fig. 1b). The velocity distribution at the inlet, v*, was first calculated in the absence of a catheter using the parabolic profile available in SimVascular (Fig. 1c). This velocity profile was used to distinguish the mesh nodes forming the vessel wall (i.e. v* = 0) from the nodes inside the vessel. For the nodes inside the vessel, the catheter radius (R), thickness (th), and eccentricity (e) which can be defined by the user were used to determine whether the mesh nodes were inside the catheter, on the catheter wall, or outside of the catheter (Fig. 1d). Besides (x,y,z) and v*, other inputs of the pipeline include the inlet blood flow rate, and catheter flow rate, geometry (radius and wall thickness), and eccentricity.

To calculate the velocity profile in the inlet plane, we assumed that blood is incompressible and Newtonian (consistent with the current version of SimVascular), and that there is a no-slip condition at the inner and outer surface of the catheter wall and vessel wall. Considering that the vessel cross-section is circular at the inlet of the computational domain and the catheter centerline is parallel to the vessel centerline (i.e. there is no catheter tip angulation), the blood velocity profile should be calculated in an annulus between the vessel wall and the catheter. We also assumed that the vessel and catheter are long enough that the blood flow is fully developed and laminar. The inlet velocity profile consisting of the catheter flow and the blood flow (u) was then calculated as follows:

- The velocity was set to zero for the nodes on the vessel wall and the catheter wall.
- The velocity profile inside the catheter can be set to zero, constant, or parabolic shape (fully-developed) depending on the application.
- To calculate the velocity distribution outside of the catheter, the mesh nodes were first mapped into a rectangle in the complex plane using a bipolar transformation. In the bipolar system, the catheter and vessel are represented by lines of constant τ where (τ,σ) are the bipolar coordinates (see Additional file 1). The velocity profile, u, was then calculated based on the solution techniques employed in the previous studies [14, 15].

### Results and discussion

It is worth noting that the main goal of the current study is to provide a pipeline to generate inlet boundary conditions for SimVascular simulations. Therefore, the sole purpose of any mesh and velocity profile presentations are to showcase examples of our pipeline outputs rather than CFD simulations and their results.

#### Sample inlet boundary conditions

Figure 1a shows a sample computational domain which represent the trunc of the hepatic artery for a cancer patient. The proposed pipeline in this study was used to calculate the velocity distribution and generate the SimVascular boundary condition file at the inlet of this geometry illustrated in Fig. 1b. Figure 1e and f show the resulting velocity distributions for a catheter wall thickness of zero with eccentricities of 0.1 and 1 mm. In this example, the inlet and catheter radii are ~ 2.25 and 1 mm (= 6 French), respectively. In both cases, it was assumed that there is no catheter flow. The effect of coarse mesh node distribution on the velocity profile interpolation can be seen inside the catheter where the velocity should be zero (dark blue) based on the zero-velocity assumption. In addition, higher-velocity regions (red areas) have formed in Fig. 1f where the catheter eccentricity is larger than Fig. 1e.

The catheter wall thickness and radius can be adjusted in our pipeline to model different types of catheters. Figure 1g, h, and e show the velocity distribution for an arbitrary catheter wall thickness of zero, 0.1, and 0.2 mm, respectively. To enforce no flow at the mesh nodes within the catheter wall in the inlet cross-section, the velocity at these nodes was set to zero. However, in these cases, the number of mesh nodes was not sufficient for a smooth circular zero velocity inside the catheter (and on its wall).

#### Mesh density effect

The finer the mesh is, the more accurate the velocity interpolations are. For example, the zero velocity profile inside the catheter is better estimated in Fig. 1j with a finer mesh compared to Fig. 1g. In addition, more nodes resided on the catheter wall in Fig. 1k compared to Fig. 1h, which resulted in a more accurate presentation of the zero velocity condition (i.e. no flow) on the catheter wall. The effect of mesh density on the calculated velocity profile can be seen in other areas of the inlet too. Table 1 lists the number of mesh nodes in the inlet plane used to generate the sample outputs of our pipeline presented in this study.

**Table 1:** Number of mesh nodes in the inlet plane used to generate sample inlet boundary conditions

Number of mesh nodes	114	310	4271
Sample output	Fig. 1e Fig. 1f Fig. 1g Fig. 1h Fig. 1i	Fig. 1j Fig. 1k Fig. 1l Fig. 3 Additional file 3	Additional file 2

#### Catheter flow

In many applications, the catheter is inserted into the vessel for drainage or administration of fluids and gasses [16–19]. Therefore, the assumption of no catheter flow is not always true and it is necessary to model the catheter in- or out-flow (positive or negative flow, respectively, according to SimVascular convention). SimVascular does not treat different inlet flow regimes (e.g. positive or negative) differently. In case, there is backflow at some outlets due to a catheter in-flow condition at the inlet, it will be addressed by SimVascular’s backflow stabilization [20].

In Fig. 1l, the catheter out-flow (i.e. forward flow) is modeled with a parabolic velocity profile. Although the fine mesh used in this case was good enough in most regions especially when compared with Fig. 1i, a finer mesh with still higher local density around the catheter wall would result in a better inlet velocity profile at the catheter boundaries (e.g. see the light blue region bridged inside and outside of the catheter). Additional file 2 presents a smoother velocity distribution for a finer mesh.

### Sample application

**Fig. 3:**
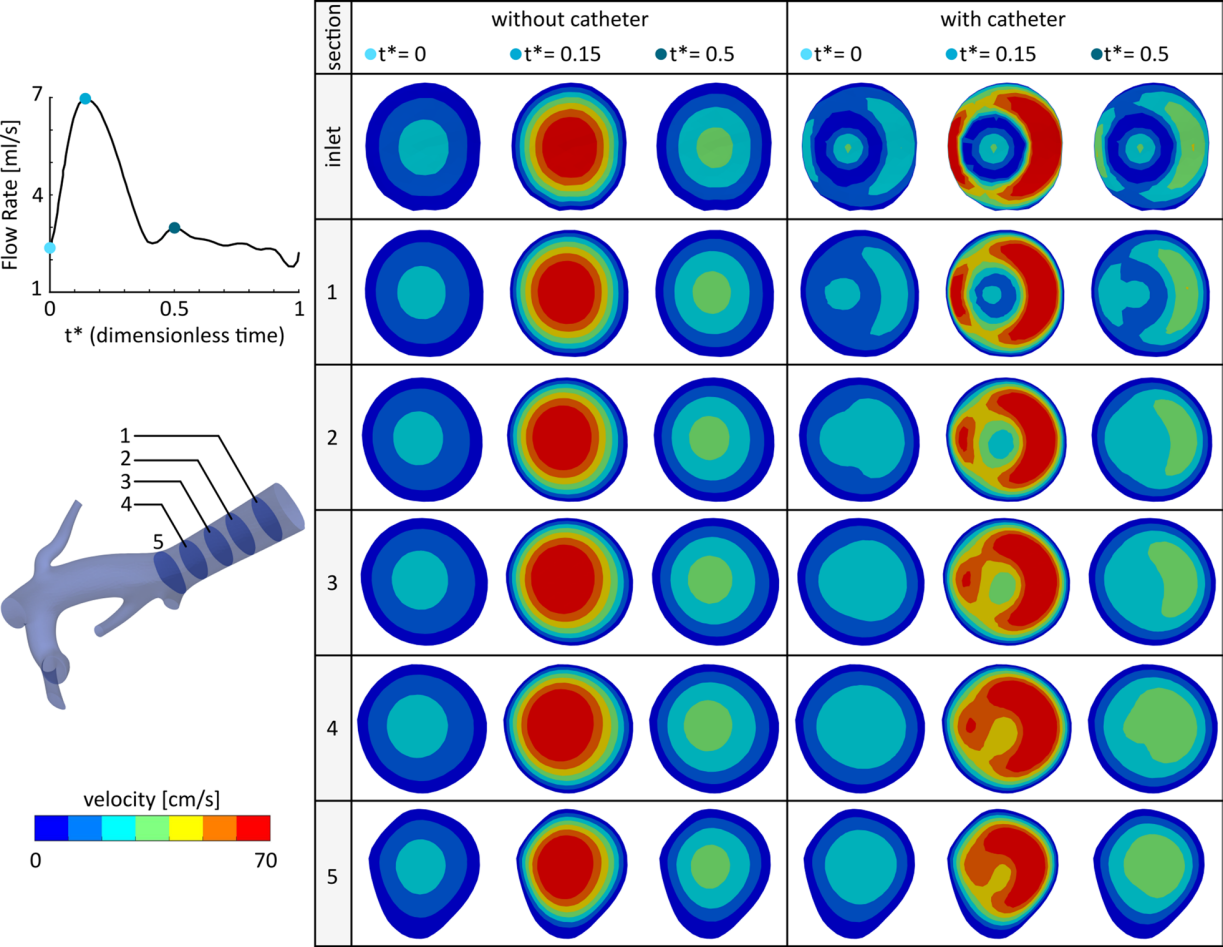
Sample application of the developed pipeline to investigate the effect of catheter presence at the inlet on the downstream velocity distribution

Yttrium-90 (Y-90) radioembolization is a form of brachytherapy to treat liver cancer where Y-90 microspheres are infused into the hepatic artery through a catheter. Computer modeling of the hepatic hemodynamics can help investigate the distribution of Y-90 microspheres in the liver. In this study, the inlet boundary condition generated with the proposed pipeline was used to qualitatively investigate the effect of a catheter on the hepatic blood flow in a patient with hepatocellular carcinoma. The computational domain consisted of the trunk of the right hepatic arterial tree (Fig. 1a) and was segmented from the cone-beam CT scans of the patient. A pulsatile flow was assigned at the inlet, Fig. 3 (available in [13]). The CFD simulations were carried out in the presence and absence of a catheter at the inlet plane. The catheter had a radius of 1 mm, a wall thickness of 0.2 mm, and an eccentricity of 0.5 mm (Fig. 1l). The catheter flow rate was arbitrarily chosen to be 0.5 mL/s with a parabolic velocity profile. More details on the methods and assumptions of the computational model preparation and CFD simulations are provided in Ref. [21, 22].

Velocity distributions are shown in Fig. 3 for five cross-sections from the inlet to the first bifurcation at three different time points over a cardiac cycle (An additional movie file shows this in more detail [see Additional file 3]). In the absence of a catheter, the velocity profile remained almost parabolic from the inlet to cross-section 5 during the cardiac cycle, as shown by the first three columns. In contrast, the catheter presence affected the velocity distribution downstream of the inlet. At lower flow rates such as t* = 0 or 0.5, the downstream flow reached a fully-developed state along the initial part of the arterial tree (before the first bifurcation). For example, at t* = 0, the flow demonstrated a parabolic shape from cross-section 3. However, the catheter effect can be clearly seen until cross-section 5 for higher flow rates such as at t* = 0.15.

## Limitations

In this study, we developed a pipeline to model the catheter presence in CFD simulations carried out with SimVascular. Due to the functionalities currently available in the software (version: September 2020), we could not define different material properties such as density and dynamic viscosity for the catheter flow compared to the fluid that flows outside of the catheter (e.g. blood).

To calculate the velocity distribution outside of the catheter, we assumed that the vessel has a circular cross-section at the inlet, and then utilized the method provided in the previous studies [14, 15] to find the exact solution for the flow between two eccentric cylinders. Therefore, any deviations of the inlet from a circular shape will introduce inaccuracies in the calculated velocity distribution especially near the vessel wall. In addition, the catheter axis is assumed to be parallel to the vessel centerline, and thus the current method cannot model any catheter tip orientations. In summary, the proposed pipeline help model the flow field downstream of the inlet more accurately when there is a catheter at the inlet in the open-source software SimVascular.

## Supplementary Information

**Additional file 1.** Velocity distribution formulation. The analytical solution used to determine the velocity distribution in the eccentric annulus formed between the vessel wall and catheter.

**Additional file 2.** Inlet condition with fine mesh. Sample output of the developed pipeline with a finer mesh density compared to those ones presented in Fig. 1. In this case, a 2.4 F microcatheter with the inner and outer diameter of 0.57 and 0.8 mm, respectively, is modeled. The administration flow rate and eccentricity were 0.33 ml/sec and 0.5 mm, respectively.

**Additional file 3.** Sample application. Sample application of the developed pipeline to investigate the effect of catheter presence at the inlet on the downstream velocity distribution.

## Data Availability

The SimVascular Development project as well as the datasets generated and analysed during the current study are available under MIT license in the following GitHub repository, https://github.com/mirtatae/simvascularDevelopment [13]. This pipeline is programmed in MATLAB and is independent of the operating system platforms. The current version at the time of this publication (v1.01) is archived in [23].

## Abbreviations

CFD: Computational fluid dynamics
CT: Computed tomography
e: Catheter eccentricity (distance between the catheter and vessel centers)
R: Catheter radius
th: Catheter wall thickness
t*: Dimensionless time
u: Inlet velocity profile in the presence of the catheter
v*: Inlet velocity in the absence of the catheter
(x,y,z): Cartesian coordinates of the mesh nodes in the inlet plane
