# Supplementary material for: Realistic boundary conditions in SimVascular through inlet catheter modeling
Source: BMC Res Notes. 2021 May 31;14:215. doi: 10.1186/s13104-021-05631-7 (PMC8186195; doi:10.1186/s13104-021-05631-7)
Supplement: Supplementary file 1 — Additional file 1. Velocity distribution formulation. The analytical solution used to determine the velocity distribution in the eccentric annulus formed between the vessel wall and catheter. [file 13104_2021_5631_MOESM1_ESM.pdf]

ADDITIONAL FILE 1

Realistic boundary conditions in SimVascular through inlet catheter modeling

Amirtahà Taebi[1], Selin Berk[2], Emilie Roncali[1,3]

[1] Department of Biomedical Engineering, University of California, Davis, One Shields Ave., Davis, CA 95616, USA

[2] Department of Mechanical and Aerospace Engineering, University of California, Los Angeles, 420 Westwood Plaza, Los Angeles, CA 90095-1597

[3] Department of Radiology, University of California, Davis, 4860 Y Street, Suite 3100, Sacramento, CA 95817, USA

Introduction

In this study, the velocity distribution outside of the catheter is calculated based on the analytical solution provided in the previous studies [1, 2]. A brief overview of this solution is described here.

Methods

To calculate the velocity distribution outside of the catheter, the following assumptions were made:

- The catheter centerline is parallel to the arterial centerline.
- The vessel cross-section is circular. Therefore, the vessel and catheter form an eccentric annulus (Fig. S1).
- The annulus is long enough that the flow becomes fully developed.

The flow in the eccentric annulus was solved using the following transformation [1]

$$x + iy = ic\,cot((\sigma + i\tau)/2) \tag{1}$$

$$c = R\sinh\alpha = r_v \sinh\beta \tag{2}$$

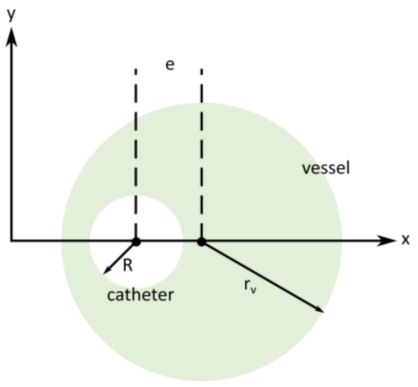

Fig. S1 Eccentric annulus formed between the vessel and the catheter.

where c is a constant, $i = (-1)^{1/2}$, (τ,σ) are the bipolar coordinates, R is the catheter radius, and r_v is the vessel radius. In this bipolar system, the catheter and vessel are represented by α and β which are lines of constant τ. These lines can be calculated as [1]

$$\cosh\alpha = (\gamma(1+\varphi^2) + (1-\varphi^2))/2\gamma\varphi \tag{3}$$

$$\cosh\beta = (\gamma(1-\varphi^2) + (1+\varphi^2))/2\varphi \tag{4}$$

$$\gamma = R/r_v \tag{5}$$

$$\varphi = e/(r_v - R) \tag{6}$$

where e is the eccentricity. Considering the boundary conditions [2], the general solution of the equation of motion for fully-developed laminar flow may be written as [1]

$$u = F + E\tau - \coth\tau/2 + \sum_{n=1}^{\infty}\{A_n e^{n\tau} + (B_n - \coth\tau)e^{-n\tau}\}\cos n\sigma \tag{7}$$

where u is the dimensionless velocity in the eccentric annulus between the vessel and the catheter, and

$$F = (\alpha\coth\beta - \beta\coth\alpha)/2(\alpha-\beta) \tag{8}$$

$$E = (\coth\alpha - \coth\beta)/2(\alpha-\beta) \tag{9}$$

$$A_n = (\coth\alpha - \coth\beta)/(e^{2n\alpha} - e^{2n\beta}) \tag{10}$$

$$B_n = (e^{2n\alpha}\coth\beta - e^{2n\beta}\coth\alpha)/(e^{2n\alpha} - e^{2n\beta}) \tag{11}$$

References

1. Snyder WT, Goldstein GA. An analysis of fully developed laminar flow in an eccentric annulus. AIChE J. 1965;11:462–7. doi:10.1002/aic.690110319.

2. Kolitawong C, Giacomin AJ. Axial flow between eccentric cylinders. Polym Plast Technol Eng. 2001;40:363–84. doi:10.1081/PPT-100000254.
