# Supplementary figures and images for: Realistic boundary conditions in SimVascular through inlet catheter modeling
Source: BMC Res Notes. 2021 May 31;14:215. doi: 10.1186/s13104-021-05631-7 (PMC8186195; doi:10.1186/s13104-021-05631-7)

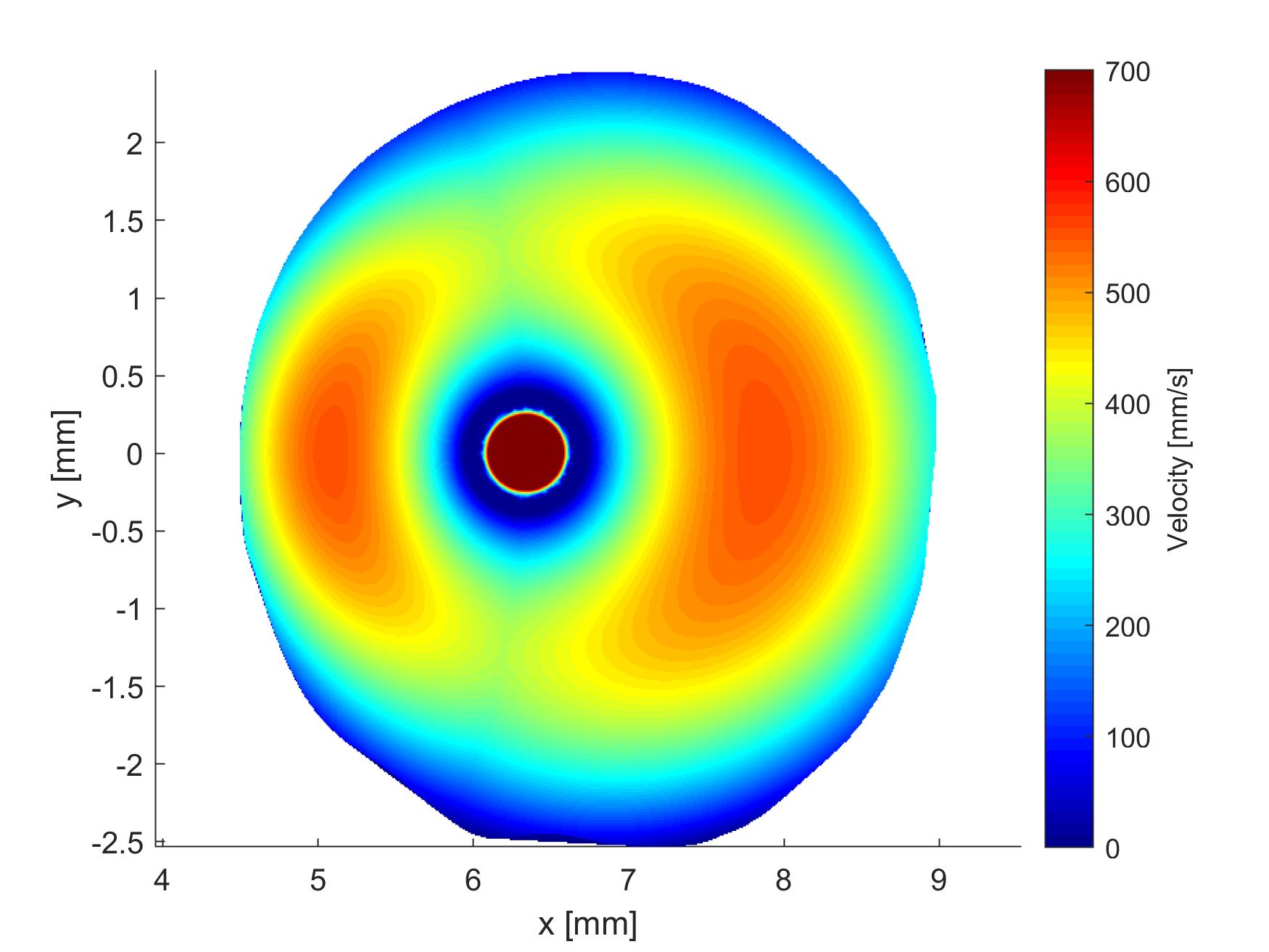

Supplement: Supplementary file 2 — Additional file 2. Inlet condition with fine mesh. Sample output of the developed pipeline with a finer mesh density compared to those ones presented in Fig. 1. In this case, a 2.4 F microcatheter with the inner and outer diameter of 0.57 and 0.8 mm, respectively, is modeled. The administration flow rate and eccentricity were 0.33 ml/sec and 0.5 mm, respectively. [file 13104_2021_5631_MOESM2_ESM.jpg]

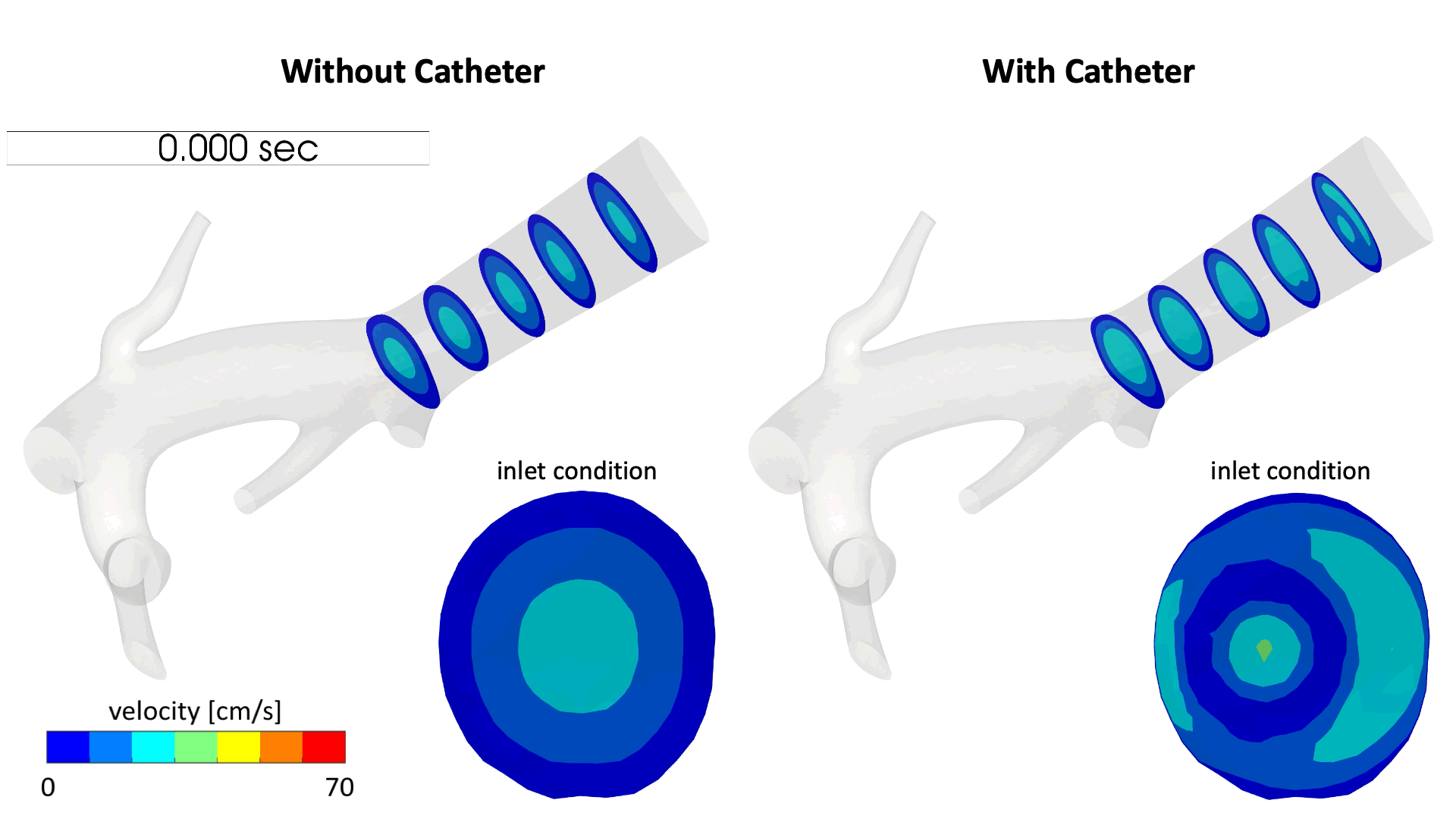

Supplement: Supplementary file 3 — Additional file 3. Sample application. Sample application of the developed pipeline to investigate the effect of catheter presence at the inlet on the downstream velocity distribution. [file 13104_2021_5631_MOESM3_ESM.gif]
